# Using the cell phone while standing or walking affects balance and mobility in people with Parkinson's disease

**DOI:** 10.1055/s-0043-1767825

**Published:** 2023-05-09

**Authors:** Tayla Borges Lino, Milena Nunes de Oliveira da Silva, Isabela Corrêa de Paula, Sarah Jane Lemos de Melo, Suzi Rosa Miziara Barbosa, Gustavo Christofoletti

**Affiliations:** 1 Universidade Federal de Mato Grosso do Sul, Faculdade de Medicina, Campo Grande MS, Brazil.; 2 Universidade Federal de Mato Grosso do Sul, Instituto de Saúde, Campo Grande MS, Brazil.

**Keywords:** Parkinson Disease, Cell Phone, Attentional Bias, Mobility Limitation, Accidental Falls, Doença de Parkinson, Telefone Celular, Viés de Atenção, Limitação da Mobilidade, Acidentes por Quedas

## Abstract

**Background** Cell phones are part of peoples' lives. The literature indicates risks when cell phones are used during a secondary motor task. Studies addressing this topic in people with Parkinson's disease are still scarce.

**Objective** To investigate the impact of daily dual tasks with cell phone on balance and mobility in people with Parkinson's disease, compared to healthy control peers.

**Methods** Participants with Parkinson's disease and controls underwent three motor tasks: (1) Standing and walking without using a cell phone; (2) Standing and walking while talking on the phone; and (3) Standing and walking while texting messages on the phone. Assessments involved balance and mobility tests. Statistical analysis was performed with multivariate analysis of variance, comparing main effect for group (Parkinson's disease × control), task (using × not using cell phone) and interactions (group × task). Significance was set at 5%. Effect sizes are reported.

**Results** Participants with Parkinson's disease showed worse balance ( *p* = 0.001, effect size of 0.471) and mobility ( *p* = 0.001, effect size of 0.472) than control peers. The use of cell phone while performing a secondary motor task affected both groups ( *p* = 0.005, effect size of 0.673 for balance and *p* = 0.001, effect size of 0.549 for mobility). The dual task impact, however, was higher in the Parkinson's disease group ( *p* = 0.009, effect size of 0.407 for mobility).

**Conclusion** Daily dual tasks with cell phones increase imbalance and mobility risks in Parkinson's disease. People should be careful when using their cell phone while standing or walking.

## Resumo

**Antecedentes** Aparelhos celulares fazem parte da vida das pessoas. A literatura aponta riscos quando o uso do celular está associado a uma tarefa motora. Estudos abordando esse tema na doença de Parkinson são escassos.

**Objetivo** Investigar o impacto de tarefas-duplas com o celular sobre equilíbrio e mobilidade de pessoas com doença de Parkinson, na comparação com controles saudáveis.

**Métodos** Participantes com e sem doença de Parkinson foram submetidos a três tarefas: (1) Ficar em pé e caminhar sem o celular; (2) Ficar em pé e caminhar enquanto conversa ao celular; e (3) Ficar em pé e caminhar enquanto digita mensagens. As avaliações envolveram testes de equilíbrio e mobilidade. Os procedimentos estatísticos envolveram testes de análise múltipla de variâncias, com análise de efeito principal para os fatores grupo (doença de Parkinson × controle), tarefa (com celular × sem celular) e interação (grupo × tarefa). Significância foi estipulada em 5%. Tamanhos de efeito foram reportados.

**Resultados** Participantes com doença de Parkinson apresentaram pior equilíbrio ( *p* = 0,001; tamanho do efeito: 0,471) e mobilidade ( *p* = 0,001; tamanho do efeito: 0,472) que controles. O uso do celular afetou ambos os grupos ( *p* = 0,005, tamanho do efeito de 0,673 para equilíbrio e *p* = 0,001, tamanho do efeito de 0,549 para mobilidade). O impacto da tarefa-dupla, contudo, foi maior no grupo Parkinson ( *p* = 0,009; tamanho do efeito de 0,407 para mobilidade).

**Conclusão** Tarefas simultâneas com o celular causam desequilíbrio e problemas de mobilidade na doença de Parkinson. As pessoas devem ter cuidado ao utilizar celulares durante atividades em pé e ao caminhar.

## INTRODUCTION

Parkinson's disease (PD) is known for its motor dysfunctions. The disease is caused by a cascade of apoptosis in the midbrain that ends up affecting dopaminergic neurons 1_._ Due to its connection to basal ganglia, several motor areas are affected and patients' independence gets compromised. 2

Parkinson's disease can affect younger and older adults. The disease, however, is much more common in advanced ages. 3 That means that, besides the motor deficits caused by the disease, people with PD face age-related changes.

Motor dysfunctions impact innumerous aspects of the patient's life, such as risk of falling, postural imbalance, and mobility impairment. 4 5 The loss of automaticity is a key deficit in PD. 6 With the progression of the disease, patients present difficulties in performing simultaneous tasks, making ordinary activities a real challenge. 7

The ability to perform more than one task at the same time is called dual task. Previous studies have reported difficulties of PD patients during dual tasks. 8 9 10 Most of these studies, however, assessed dual task on non-daily activities, such as walking while subtracting numbers or walking while telling the days of the week. 11

A frequent dual task performed nowadays involves cell phones. It is common to see people talking or sending messages on the phone while standing, walking, shopping, or even at the gym. So far, most of the studies that have evaluated the dual task effect of cell phones involved younger adults. 12 This probably happened because younger adults are the segment of society that has most incorporated technology in their lives. 13

Some studies assessed the dual task effect in older adults and confirmed increasing imbalance and mobility problems while using a cell phone. 14 15 16 Studies addressing this topic in neurodegenerative conditions, such as PD, are still scarce.

The aim of this study was to investigate the impact of daily dual tasks involving the use of a cell phone (texting messages and talking while standing or walking) on balance and mobility in people with PD. The researchers' hypothesis was that people with PD are subject to greater risk of falling when using their cell phones while performing a simultaneous motor task. Compared with control peers, authors expected that PD patients would present worse balance and mobility.

## METHODS

Thirty participants were enrolled in this study. The subjects were divided into the PD and control groups. The research was conducted at the applied biomechanics laboratory of Universidade Federal do Mato Grosso do Sul.

All participants provided written consent prior to assessments. Ethical approval was obtained with the institutional ethics committee (protocol # 4.062.787, CAAE # 31698120.2.0000.0021).

The sample size was calculated assuming type 1 error of 5%, power of 90%, and a Cohen d dual task effect of 1.27. The dual task effect was presented by Freitas et al. 17 when comparing manual dexterity in people with PD during a simultaneous motor task. The analysis indicated a minimum of 30 participants, with 15 in each group.

Participants with PD were selected at the neurologic outpatient clinic of the Hospital Universitário Maria Aparecida Pedrossian (Campo Grande, MS, Brazil). Subjects of the control group were recruited in the community.

The inclusion criteria for the PD group involved patients with a minimum age of 60 years, sedentary (defined as having routine activities of up to 2.0 Metabolic Equivalent of Task), not using any assistive device for locomotion, who had staged up to IV on the Hoehn Yarh scale, 18 and with a PD diagnosis according to the United Kingdom Parkinson's Disease Society Brain Bank Clinical Diagnostic Criteria. Healthy control peers had their selection criteria matched with sociodemographic characteristics of the PD group.

Cognitive decline, cerebellar dysfunction, dizziness, recent surgery (< 6 months), and the presence of other neurologic conditions were defined as exclusion criteria for both groups. Illiterate subjects, participants that never have used cell phones and those that did not have one were also excluded.

### Procedures

The methodological procedures are reported according to the Strengthening the Reporting of Observational studies in Epidemiology (STROBE) statement checklist. Assessments involved initially a sociodemographic questionnaire containing general aspects, such as sex (men or women), age (years), schooling (years), weight (Kg), height (m), body mass index (Kg/m ^2^ ), years of using cell phone, and daily hours of cell phone use. Then, the authors applied two cognitive 19 20 and one functional 21 test. All instruments used in this study are validated for the Portuguese language and are suitable to be applied in people with PD and in healthy peers.

The Mini-Mental State Exam (MMSE) 19 and the Frontal Assessment Battery (FAB) 20 assessed subjects' cognition. The MMSE addressed the following cognitive skills: temporal orientation, spatial orientation, registration of words, attention and calculation, immediate and delayed recall, language and visual-constructive practice. The MMSE score ranges from 0 to 30 points, and the cutoff points was adopted according to the recommendations in the study by Brucki et al. 22

The FAB addressed other cognitive skills, such as concept recognition, lexical flexibility, motor programming, conflicting instructions, inhibitory control, and environmental autonomy. The FAB score ranges from 0 to 18 points, and the cutoff points adopted in this study were those established by Beato et al. 23

The Pfeffer index 21 was used to assess patients' functionality. This 10-item instrument evaluates subjects' independence in performing instrumental activities of daily living. Each item is scored on a scale of 0 (independence) to 3 (dependence), and higher scores reflect greater dependency of the subject. The authors opted to use the Pfeffer index instead of other function tests because of its high specificity in distinguishing the impact of cognitive processes on activities of daily living. 24

The impact of cell phone use on mobility and balance was assessed in two motor tests. In the first test (static test), subjects were asked to stand barefoot on a force platform for 60 secs. The Biomec400 force platform (EMG System do Brasil, São José dos Campos, SP, Brazil) sampled data at 100 Hz. For the second motor test (dynamic test), subjects performed the Timed-Get-Up-and-Go (TUG) test 25 in a 5 m wide non-slip hall.

The variables assessed in the static test were limb direction (cm), imbalance speed (cm/s), and center of pressure (cm ^2^ ). Higher values indicate worse balance. The data were processed using the MATLAB program (MathWorks Inc., Natick, MA, USA) associated with a second-order digital low-pass Butterworth filter. The Biomec400 force platform was chosen due to its ability to analyze participants' center of gravity on static basis.

The variables assessed in the dynamic test were time and number of steps. The TUG measures the ability of an individual to get up from a chair, walk 3 m, come back, and sit on the chair. A large number of steps and great amount of time indicate mobility problems. The TUG test was chosen due to its ability to analyze participants' mobility and risk of falls on a dynamic basis. A 2D digital camera recorded data of the tasks.

The subjects performed both tests with and without a cell phone. The order of the tests (static × dynamic) and the tasks (with cell phone × without cell phone) themselves were random, seeking to avoid any learning effect. In the situation without the use of cell phone, the tests were performed without any distractor. In situations involving the use of cell phone, participants were instructed to put the device in their front pocket, and then perform the talking or typing tasks.

One researcher stood beside the subjects (alert in case of falls) and a second researcher stood outside the laboratory to call the participants or send a message. The talking activity involved general questions such as food preferences, sport interests, political spotlights, etc. For the sending message task, the participants were instructed to send the following text: “Good morning, I am going to be late for our appointment.” The sentence was told to the participant at the beginning of the task.

All tests were performed with participants' own cell phone. The authors opted to use the subjects' personal cell phones to avoid any delay in adapting to a new device. Writing or talking errors during dual tasks were not considered.

Complementary information addressed the clinical profile of the PD group. Disease severity was measured in time since diagnosis and on the Hoehn Yarh scale. 18 Motor impairment was assessed with the Unified Parkinson's Disease Rating scale (UPDRS). 26 The anti-PD medication was collected as per the Levodopa Equivalent Daily Dose. 27 All assessments were performed on the ‘on phase’ of the anti-PD medication.

### Statistical analysis

Statistical procedures were performed on a descriptive and inferential basis. The characterization of the groups was done by number of events for categorical variables and mean ± standard deviation for continuous variables.

At first, the authors used the Student t- and chi-squared tests to compare groups in terms of sociodemographic aspects, cognition, and functional independence. Then, the multivariate analysis of variance, applied in association with the Wilk Lambda test, provided group (PD × control), task (no cell phone × using the phone), and interaction (group × task) comparisons.

Contrast analyses were used to investigate which task was more challenging to the participants (no cell phone × texting message × talking on the phone). Significance was set at 5%. The effect sizes and statistical power are reported.

## RESULTS

Thirty participants were divided into two groups: PD and control. The groups were similar as for sample size, sex, age, schooling time, weight, height, body mass index, cognition, and years of using cell phone. Participants of the control group spend more hours per day on the phone than subjects of the PD group. PD patients showed higher scores on Pfeffer index than healthy peers. Socio-demographic characteristics of both groups and clinical aspects of the PD group are presented in Table 1 .

**Table 1:** Sociodemographic and clinical factors of the Parkinson and control groups

Variables	Groups		95% CI	*P* -value
	Parkinson	Control		
Number of participants, n	15	15	---	0.999
Sex, n (men:women)	5:10	4:11	---	0.690
Age, years	66.8 ± 13.4	67.0 ± 9.1	-8.7 to 8.5	0.975
Schooling, years	6.9 ± 2.3	7.2 ± 1.8	-1.8 to 1.2	0.662
Weight, Kg	62.6 ± 14.1	71.0 ± 12.	-18.2 to 1.5	0.094
Height, m	1.6 ± 0.1	1.6 ± 0.1	-0.1 to 0.1	0.699
Body mass index, kg/m ^2^	25.1 ± 5.7	27.6 ± 3.5	-5.9 to 1.1	0.175
Years of using cell phone	9.7 ± 6.3	8.8 ± 4.0	-3.1 to 4.8	0.660
Daily hours of cell phone use	1.4 ± 0.8	3.0 ± 1.3	-2.5 to -0.8	0.001
Mini-mental state exam, pts	25.3 ± 4.1	26.6 ± 2.7	-3.8 to 1.3	0.327
Frontal assessment battery, pts	13.2 ± 3.8	14.4 ± 2.3	-3.6 to 1.2	0.313
Pfeffer index, pts	6.0 ± 7.1	0.2 ± 0.7	2.0 to 9.6	0.007
Time since diagnosis, years	8.6 ± 7.7	---	---	---
Hoehn & Yarh scale, points	2.7 ± 1.1	---	---	---
UPDRS motor section, points	18.4 ± 9.2	---	---	---
Levodopa Equivalent Daily Dose, mg	622.7 ± 636.3	---	---	---

Abbreviations: CI, confidence interval; UPDRS, Unified Parkinson's Disease Rating Scale.

Notes: Data are presented in number of events for categorical variables and mean ± standard deviation for continuous variables. *P* -value of the chi-squared test for the categorical variables and *p* -value of the student t-test for the continuous variables.

Table 2 details the variables assessed on the force platform. Multivariate analysis of variance indicated that PD patients have worse static balance than control peers ( *p* = 0.001, group effect of 0.471). The use of cell phone while performing a secondary motor task affected both groups ( *p* = 0.005, task effect of 0.673) in a similar way ( *p* = 0.239, group × task effect of 0.430). Contrast analyses showed that standing and talking on the phone affect balance in a greater way than just standing or standing and texting message. Significant difference was seen for frontal direction ( *p* = 0.003), lateral direction ( *p* = 0.004), and frontal speed ( *p* = 0.024). No difference was seen for center of pressure ( *p* = 0.100) and lateral speed ( *p* = 0.119).

**Table 2:** Participants' balance while performing static motor tasks with and without cell phone

Balance	Groups	Tasks			MANOVA main effect		
		No cell phone	Texting message	Talking on phone	Group	Task	Group × task interaction
Frontal direction, cm	PD	4.0 ± 3.8	5.4 ± 4.5	5.8 ± 3.6	*P =* 0.001 ES = 0.471 Power = 91.0%	*p* = 0.005 ES = 0.673 Power = 95.7%	*p* = 0.239 ES = 0.430 Power = 51.4%
	Control	2.7 ± 1.5	3.1 ± 1.5	4.3 ± 2.7			
Lateral direction, cm	PD	5.0 ± 6.0	5.6 ± 4.6	7.4 ± 5.3			
	Control	1.5 ± 1.0	1.5 ± 0.8	2.4 ± 2.0			
Frontal speed, cm/s	PD	3.2 ± 4.3	3.8 ± 3.9	4.4 ± 4.0			
	Control	1.2 ± 0.4	1.4 ± 0.3	1.8 ± 0.6			
Lateral speed, cm/s	PD	3.5 ± 6.0	4.2 ± 5.9	4.9 ± 5.5			
	Control	1.1 ± 0.3	1.2 ± 0.3	1.5 ± 0.3			
Center of pressure, cm ^2^	PD	20.6 ± 50.4	34.3 ± 82.9	32.1 ± 45.5			
	Control	2.6 ± 2.1	3.4 ± 2.6	7.3 ± 6.4			

Abbreviations: ES, effect size; MANOVA, multivariate analysis of variance; PD, Parkinson's disease.

Notes: Data are expressed in mean ± standard deviation. *P* -value, effect size and power analyses of the multivariate analysis of variance. Contrast analyses showed that standing and talking on the phone affect balance in a bigger way than just standing or standing and typing messages on the phone.

Table 3 details the performance of the participants on the TUG test. Multivariate analysis of variance indicated that PD patients had worse mobility than the subjects of the control group ( *p* = 0.001, group effect of 0.472). The use of cell phone while walking affected mobility in both groups ( *p* = 0.001, task effect of 0.549). The impact of dual tasking with cell phone, however, was higher in the PD group ( *p* = 0.009, group × task effect of 0.407). Contrast analysis showed that texting messages resulted in greater changes in the time and number of steps relative to the talking and no cell phone conditions ( *p* = 0.026 for time and *p* = 0.015 for number of steps).

**Table 3:** Performance of the participants on the Timed-Get-Up-and-Go test

Timed-Get Up-and-Go test	Groups	Tasks			MANOVA main effect		
		No cell phone	Texting message	Talking on phone	Group	Task	Group × task interaction
Time, sec	PD	18.9 ± 9.3	51.0 ± 34.3	27.0 ± 10.9	*p* = 0.001 ES = 0.472 Power = 99.0%	*p* = .001 ES = 0.549 Power = 99.0%	*p* = 0.009 ES = 0.407 Power = 87.2%
	Control	11.2 ± 2.5	15.8 ± 5.1	13.6 ± 3.9			
Steps, n	PD	24.1 ± 7.2	48.9 ± 24.3	32.7 ± 12.5			
	Control	15.1 ± 3.9	18.4 ± 4.0	17.2 ± 3.7			

Abbreviations: ES, effect size; MANOVA, multivariate analysis of variance; PD, Parkinson's disease.

Notes: Data are expressed in mean ± standard deviation. *P* -value, effect size and power analyses of the multivariate analysis of variance. Contrast analyses showed that walking and texting message affect mobility in a bigger way than just walking or walking and talking on the phone.

No participant experienced any discomfort during the tests. The subjects performed all the tests accordingly, without falling down or dropping their cell phones.

## DISCUSSION

This study verified the influence of daily dual-tasks involving the use of cell phone on balance and mobility in people with PD. The results suggest that PD patients have worse balance and mobility than control peers. In both groups, texting messages was more challenging while walking. Talking on the phone, differently, was more challenging while standing. We present here the discussion of the findings, which can be of great importance to patients, family members, and health care professionals.

The results presented in Table 1 confirm that the groups were homogeneous in terms of sociodemographic aspects and cognition. This is particularly important for cognition, knowing that cognitive decline is highly prevalent in PD. 28 Other factors, such as age and schooling, could also bias the results. Table 1 reinforces that those aspects were controlled, and they did not influence the results of this study.

Differences between groups were seen for the Pfeffer index and for daily use of cell phone. Dutra et al. 29 reported the cutoff point higher than three as an indicator of functional impairment. In this study, the PD group presented a considerable degree of impairment, with a mean score of 6 pts. We believe that patients' scores in the Pfeffer index is associated with a lower cell phone use of the PD group, since the instrument assesses specific activities that require manual dexterity (also necessary for handling cell phones).

Table 1 shows that the PD group was formed by subjects with a mean diagnosis time of 8 years, mean score of ∼ 3 pts on the Hoehn & Yarh scale and 18 pts on the motor section of the UPDRS. The participants' levodopa equivalent daily dose was of 622.7 mg. These data confirm that the results are restricted to subjects in the moderate stage of the disease and on the “on phase” of their anti-PD medication.

Previous studies report the impact of PD on subjects' balance. 30 31 32 The disability is caused by apoptosis of neurons in the basal ganglia (nigro-striatal and dentato-pallidal pathways), which ends up affecting the cerebellum and other motor areas. 33 This study confirmed that balance in PD participants is more compromised than in healthy control peers. The results also corroborate previous studies by showing the impact of dual tasking in PD. 8 9 10 11

In spite of individuals with PD having a worse overall standing balance than individuals without the disease, the impact of performing an additional task was similar for both groups. In other words, PD has not potentiated the dual task effect on static balance. This finding was similar to the one reported by Fernandes et al. 34

A recent meta-analysis highlights that dual tasks severely disrupt mobility in people with PD. 8 Such a result was confirmed in the present study. Participants with PD had worse mobility than healthy subjects, and both groups were affected by task complexity.

Differently from what was observed regarding standing balance, PD potentiated the dual task effect on mobility. That is, PD participants not only presented worse mobility than control peers, but the dual task effect was higher in this group. The authors attribute the higher impact of PD on mobility than on static balance to freezing and loss of automaticity, both affecting patients while walking. 4 5 6

Few studies have addressed the dual task effect of using cell phones in PD patients. Yamada et al., 11 for example, found that talking on the phone while walking brings more risks to patients than just walking or walking and carrying bags. This confirms the impact of cognitive processes on mobility. 35

An interesting finding is that participants with PD showed more standing imbalance when talking on the phone than when texting messages or with no phone. Authors attribute the higher difficulty in performing the talking task while standing to the activation of different speech areas, such as the primary auditory cortex, the Wernicks and the Brocas areas. Since speech problems are common in PD, 36 the authors hypothesized that these areas may have centralized patient's attention, reducing the attention that should be given to the motor areas. Confirmation of this finding requires further studies.

Differently, patients with PD had more mobility problems when texting messages than when talking on the phone or just walking. The authors believe that, although the act of texting messages may be increasingly common nowadays, it is unlikely to be as well practiced as walking and talking. For Lamberg and Muratori, 37 the increased attentional demands required for texting messages may lead to errors in the otherwise subconscious task of walking. This may imply a greater cognitive effort in performing the walking and texting task.

The authors identified that all participants performed the typing task properly. This may explain the growing imbalance caused by the simultaneous task of texting message and walking. Although writing errors were not accounted for in this study, the authors observed an effort of all subjects to finish the typing task during the walking test.

This study presents three limitations. First, the results are restricted to PD patients in the moderate stage of the disease and in the “on phase” of the anti-PD medication. Assessing patients in the “off phase” would provide a better view of the impact of the disease on the use of cell phone during dual tasks. However, since the aim of this study was to investigate the impact of cell phone use on daily dual tasks, the assessment of patients in the “on phase” proved to be more appropriate.

Second, the type of cell phone might have influenced the results, since each device has its cognitive ergonomics in order to facilitate its use by participants. Third, subjects in the control group spent more hours per day using their cell phones than participants in the PD group. Despite the authors having controlled the years of cell phone use in both groups, the higher daily use of cell phone by the control group could provide a better manual dexterity with cell phones than the PD group. This finding requires further studies analyzing how manual dexterity in PD affects cell phone use by patients.

In summary, daily dual tasks with cell phones affect balance and mobility in people with PD. Texting messages was more challenging while walking, and talking on the phone was more challenging while standing. New studies with a more representative sample size should be performed to confirm these findings.
